# Problems with Evaluation of Micro-Pore Size in Silicon Carbide Using Synchrotron X-ray Phase Contrast Imaging

**DOI:** 10.3390/ma15030856

**Published:** 2022-01-23

**Authors:** Tatiana S. Argunova, Victor G. Kohn

**Affiliations:** 1 Ioffe Institute of the Russian Academy of Sciences, Polytekhnicheskaya st. 26, 194021 St. Petersburg, Russia; 2 National Research Centre ‘Kurchatov Institute’, Kurchatov sqr., 1, 123182 Moscow, Russia; kohnvict@yandex.ru

**Keywords:** micro-pores in single crystals, X-ray imaging, phase contrast, synchrotron radiation

## Abstract

We report near- and far-field computer simulations of synchrotron X-ray phase-contrast images using a micropipe in a SiC crystal as a model system. Experimental images illustrate the theoretical results. The properties of nearly perfect single crystals of silicon carbide are strongly affected by μm-sized pores even if their distribution in a crystal bulk is sparse. A non-destructive technique to reveal the pores is in-line phase-contrast imaging with synchrotron radiation. A quantitative approach to evaluating pore sizes is the use of computer simulations of phase-contrast images. It was found that near-field phase-contrast images are formed at very short distances behind a sample. We estimated these distances for tiny pores. The Fresnel zones did not provide any information on the pore size in the far-field, but a contrast value within the first Fresnel zone could be used for simulations. Finally, general problems in evaluating a micro-pore size via image analysis are discussed.

## 1. Introduction

The structural homogeneity and perfection of silicon carbide (SiC) single crystals have been improved in recent years by developing physical vapor transport technology. As a result, micropipes as hollow cylindrical pores associated with screw super-dislocations with giant Burgers vectors have been eliminated from commercial substrates. These defects are much more critical for device stability than closed-core screw dislocations because they lead to the failure of micro-plasmas in the pipes [1]. Furthermore, the current availability of zero-micropipe material allows the areas of high-voltage p-n junctions to be increased without harmful electrical consequences [2]. Nevertheless, the production costs of crystals with low defect densities imply that there is still room for homogeneity improvement in SiC. Future challenges in the growth of doped crystals and the further enlargement of the boule diameter may cause risks associated with polytype instabilities and the generation of micro-pores. Therefore, new methods are being designed at laboratories to eliminate the micropipes: growing free-spreading crystals, using special seeds with a profiled surface (see, e.g., review [3]), and obtaining SiC on Si substrates [4], which require the diagnostics of micro-pores.

Synchrotron x-ray imaging techniques have been used to study SiC crystals for many years, firstly in Bragg diffraction (topography) mode, then with the advent of third-generation synchrotron radiation (SR) sources in phase-contrast modes. In-line phase-contrast imaging (PCI) allows one to visualize micro-objects in the volume of materials if absorption contrast is weak. Other imaging techniques such as x-ray microscopy (XRM), coherent diffraction imaging (CDI), or x-ray ptychography can provide nano-level resolution. However, they have a relatively limited field of view. For example, XRM uses focusing devices: compound refractive lenses, zone plates, or multilayer mirrors [5]. CDI is based on a registration of the diffraction pattern of a nano-object in the far-field region and solving the inverse problem through computer algorithms that produce a real space image of the object [6]. CDI requires reducing the beam to a size smaller than the lateral coherence length, which typically equals ~30 µm and rarely exceeds 100 µm. x-ray ptychography retrieves the phase-related information on a larger object structure from the ensemble of CDI patterns formed during the scan [7].

We can note that XRM, CDI, and x-ray ptychography are hardly applicable for diagnostics of sparse distribution of defects such as pores, inclusions, or micro-cracks in nearly perfect crystals. Only the in-line PCI [8,9] with a large view-field is suitable for such a purpose. A wide, diverging, partially coherent SR beam makes it possible to detect the crystal defects by the total phase shift along the beam path. In this technique, image features depend on the sample-to-detector distance *z*. The real size of, say, a micro-pore in a single crystal correlates with the image size only on a very short distance behind the sample. For that, the near-field condition has to be fulfilled; namely, 2 *r*_1_ << *D*, where *r*_1_ = (*λ z*)^1/2^ is the radius of the first Fresnel zone for the wavelength *λ* and *D* is the transverse pore size. Towards the far-field region, where 2 *r*_1_ >> *D*, the fringe pattern arises, and the object size is visible only in the modulation of the fringes. Quantitative information from image data can be obtained by solving the inverse problem.

The goal of the inverse problem solution is the phase shift created by the object. Nowadays, phase mapping is customarily used to characterize objects on the micron and sub-micron scale (see, e.g., a book [10]). An alternative approach is computer simulations of experimental images. We refer to several papers dealing with different pore models [11,12,13] and reviews of recent literature [14,15].

When small pores occur during the sublimation growth of SiC single crystals, their size is an important parameter that could affect the properties of the wafer material for device fabrication. This paper presents experimental and simulated phase-contrast images corresponding to different distances behind the sample to evaluate a proper transverse size of a micro-pore from image analysis. It is of particular interest to know the maximum distance from the sample where the size of the phase-contrast image of a micro-pore equals the transverse dimension of the pore. For smaller distances, the pore diameter can be determined directly from the image pixels with a high-resolution charge-coupled device (CCD), provided that its resolution is satisfactory. However, we must conclude that the distance is too small and practically unattainable.

## 2. Experiment

SiC wafers were investigated using the in-line PCI technique at the Pohang Light Source facility operated at 3.0 GeV in Pohang, Korea. We used the x-ray micro-imaging beamline, whose bending magnet gave an effective source of small size (60 µm and 160 µm in the vertical and horizontal directions) located far away (34 m) from the sample. Therefore, the SR beam could be considered parallel, and the spatial resolution mainly resulted from the effective pixel size of the detector. The pco.4000 CCD camera (PCO Imaging, Kelheim, Germany) had 4008 × 2672 pixel resolution and 9 × 9 μm^2^ pixel size. X-rays were converted to visible light using a crystal scintillator. A light image was magnified 20× by an optical lens between the scintillator and CCD. Using a lens decreases the pixel-to-object size ratio so that image scaling “reduces” the effective pixel size to 0.45 µm. We noticed that the detector consisting of a scintillator, optical lens system, and CCD did not allow us to approach close to the sample.

We prepared a particular specimen whose surface was parallel to the <0001> growth direction. This was an (−1100) oriented slice (2.5 cm^2^ in size and 0.5 mm thickness) of 4H-SiC boule. The specimen contained micropipes, or open-core screw super-dislocations, propagated mainly in the <0001> direction. The distance between the pipes varied from a few tens to a few hundred microns. Their transverse sizes looked variable, but they retained the direction of propagation. Other micropipes deviated from the <0001> at rather large angles. For our imaging experiments, we chose several pipes lying remotely from each other at a distance of ~100 μm with the axes parallel to <0001>. The holder fixed the sample with a pipe axis horizontal and perpendicular to the beam. The CCD detector with 1804 × 1202 µm^2^ view field recorded phase-contrast images in multilayer beam mode with a typical exposure time of about 10 sec.

**Figure 1:**
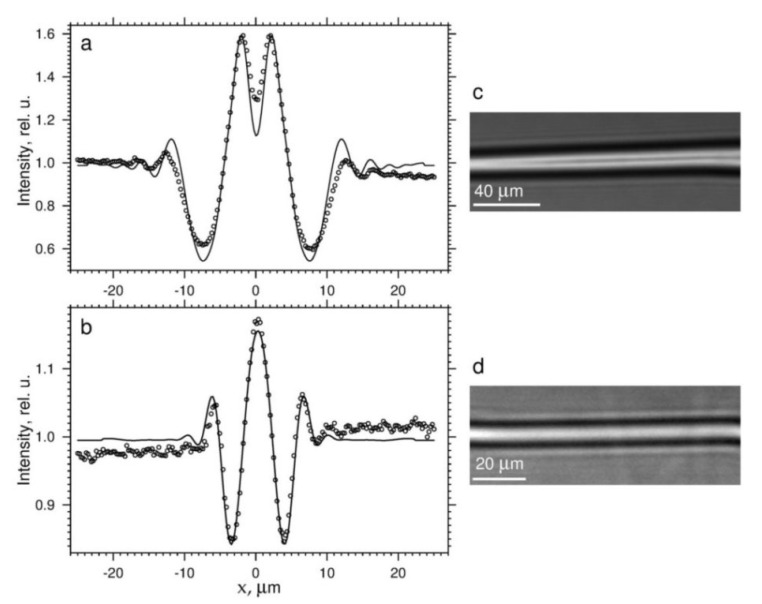
Experimental (circular markers) and theoretical (solid lines) intensity distribution across the axis of a pipe having the diameters: (**a**) *D* = 14.35 µm; (**b**) *d*_1_ = 2.41 µm (transverse) and *d*_2_ = 2.38 µm (longitudinal). See text for details. (**c**,**d**) Phase-contrast images of the thick and the thin pipe, respectively. The intensity profile was measured in the middle of each image.

The propagation distance in the near field region is related to the pore size. Let us consider two pipes located in the bulk of the sample; and let the pipes have different diameters *D* and *d*, where *D* > *d*. We determined the diameters by fitting the simulated images created by our program FIMTIM (Fit Micro-Tube Image. Please send a message to the author Kohn V.G in case of interest.) We elaborated this program to simulate phase-contrast images for a pink or monochromatic SR beam and automatically fit the experimental images [16]. The procedure was as follows. First, we measured the contrast within a 2D image region around each pipe across its axis. The FIMTIM program was used at the following experimental parameters: SR was monochromated with a multilayer mirror of the spectral resolution Δ*E*/*E* = 0.7% and for the photon energy *E* = 16 keV (λ = 0.775 Å). The specimen-to-scintillator distance was *z* = 40 cm. In Figure 1, markers represent normalized intensity profiles for the thick (a) and the thin (b) pipes. Solid lines show the best fit between the model and the data. Figure 1c,d, respectively, show the phase-contrast images of the thick and thin pipes recorded with a CCD camera.

The thick pipe had a transverse size of *D* = 14.35 µm. Comparison with the diameter of the first Fresnel zone 2 *r*_1_ = 11.14 µm showed that *D* > 2 *r*_1_. The near field condition was met, and the distance between the first order minima was approximately equal to *D* (Figure 1a). By looking at this figure, we noticed that the minima were spaced apart by about 15 µm. The conclusion was verified.

The cross-sectional diameters of the thin pipe determined by simulations were equal to *d*_1_ = 2.41 µm and *d*_2_ = 2.38 µm, where *d*_1_ and *d*_2_ corresponded to the directions across and along the beam. In this case, the inverse relationship between the Fresnel zone diameter and the transverse size was fulfilled: *d*_1_ < 2 *r*_1_. The distance between the minima was about three times larger than the real diameter *d*_1_ of the thin pipe. However, for *z* < 1.3 cm, *d*_1_ remained larger than 2 *r*_1_, thus indicating the ‘boundary’ between near field and far-field regimes.

## 3. Numerical Simulation

We elaborated a multipurpose computer program called XRWP1 to perform computer simulations of x-ray wave propagation through many objects of any setup of SR imaging beamline. The program is permanently developing using the programming language ACL [17]. The ACL is similar to Python. It is executed by the open-access program-interpreter vkACL.jar written in Java by Victor Kohn. The program XRWP1 is not ready for free download. In case of interest, please send a message to the author.

XRWP1 calculates the wave propagation in free space according to the Huygens–Fresnel principle as a convolution of the SR wave function with the Fresnel propagator. The convolution is calculated through the Fourier transform method. The direct and back Fourier transformations are used at short and medium distances. In the far-field region, only the direct Fourier transformation is sufficient due to the property of the Fresnel propagator.

All the Fourier transformations are calculated by FFT (fast Fourier transformation) method [18] using a grid of points with a constant step *a* = 0.1 μm and the number of points *N* = 2048. Images below display only part of the computational domain. The object is a small pore in a crystal. The pore is described by the transmission function. The phase-contrast theory has recently been reviewed by the authors [16], to which the reader is referred for further details.

**Figure 2:**
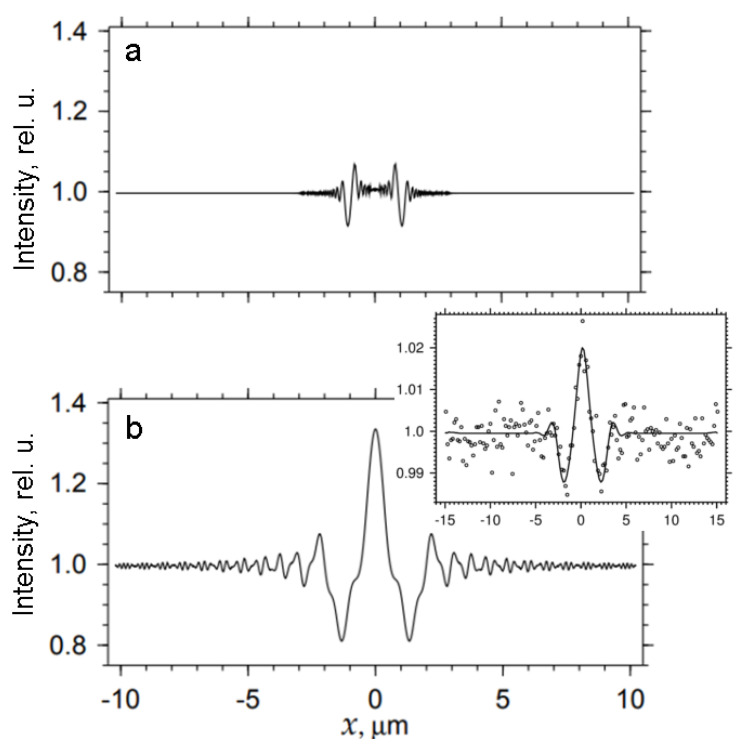
Theoretical phase contrast images of a pipe of 2 μm diameter calculated for the sample-to-detector distance *z* = 0.1 cm (**a**) and *z* = 2 cm (**b**). Inset to (b) shows experimental (circular markers) and simulated (solid line) profiles of the image of the pipe 2.79 µm in diameter recorded at *z* = 5 cm.

Figure 2a represents the theoretical image of a small-diameter pipe (2 µm) obtained by numerical simulation at a distance of 0.1 cm from a SiC specimen. Well-separated maxima and minima dominate small-scale intensity modulation, and overall image contrast is weak. Small oscillations correspond to high-order Fresnel zones, and the first Fresnel zone diameter equals 2 *r*_1_ = 0.56 µm. The picture can be explained using the geometrical optics approximation. The x-rays experience strong refraction at the pore edges because the angle between the ray’s direction and the edge is slight. As a result, the x-rays deflect to the center of the pore. They form an area of minimum intensity along its borders, whereas the highs (Figure 2a) localize in the shadow area. The distance between the deepest minima exactly equals the diameter of the pipe. The actual pipe size can be measured directly from image pixels.

A slight increase in the distance *z* (from 0.1 to 0.5 cm) makes minor changes in the pattern, and the distance between the minima still corresponds to the transverse diameter of the pipe. After that, however, the prominent intensity oscillations become wider. At the same time, the width of the whole image grows with *z*. Geometrical optics explanation of the image width is not entirely satisfactory. As is known from a single slit diffraction theory, a plane wave, having passed through the slit, acquires an angular divergence of the order of *α* = *λ*/*D*_s_, where *D*_s_ is the slit size. The maximum size of the entire image is the sum of the shadow size and the magnification part, which is directly proportional to the distance *z*. The latter term is approximately equal to *λ z*/*D*_s_. An analog to this result is known in quantum mechanics as the uncertainty relation. Replacing *D*_s_ by *D* = 2 µm in the above formula, we obtain the following result: at a distance of *z* = 0.1 cm, the magnification part equals 0.04 µm, which seems negligible. Nevertheless, at a distance of 0.5 cm, it is already five times larger.

The diameter 2 *r*_1_ remains less than the size *D* = 2 µm of the pipe to be imaged as long as *z* ≤ 1.3 cm. Another critical parameter is the distance of focusing x-rays exiting the central part of the pipe: *F* = *D*/4 *δ*, where *δ* is the refractive index decrement. In our case, *δ* = 2.6 × 10^−6^ and *F* = 1.9 cm. Figure 2b shows an image of the same pore at a *z* = 2 cm distance, when *z* corresponds to the focusing distance *F*. A single peak appears for the first time in the center of the pattern; this peak remains present for all values of z > *F*. We notice that Fresnel zones do not yet reveal a sinusoidal shape because of interference, determined by the zones located at different pore edges. One can roughly estimate the diameter of the pipe by using the first order minima, but any averaging will change the result.

The experimental intensity distribution shown in Figure 2b is generally consistent with the theoretical image. However, we have only a central maximum and two flattened side oscillations against a background of solid noise specific to broadband low-intensity radiation. First-zone oscillations allow the registration of a micropipe. At the same time, higher-order Fresnel zones are entirely suppressed. The solid line represents the theoretical curve obtained using the FIMTIM program for the sample-to-detector distance *z* = 5 cm. The pipe diameter corresponding to the best fit between the simulation and the experiment equals *D* = 2.79 µm.

**Figure 3:**
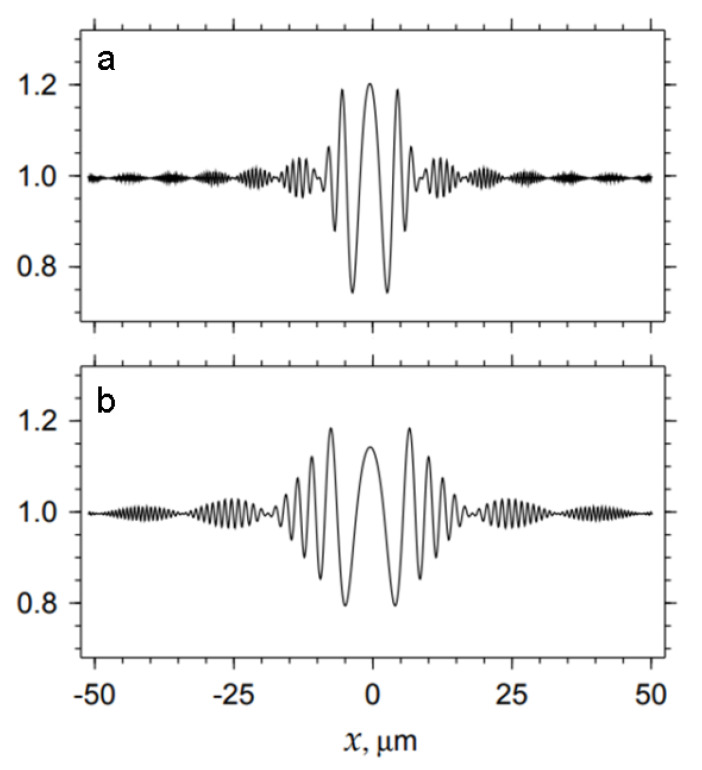
Simulated results for the intensity distribution in far field at the distances *z* = 20 cm (**a**) and *z* = 40 cm (**b**). The pipe diameter equals 2 µm.

Let us now turn to the Fraunhofer diffraction pattern of a small-diameter pipe (2 µm), which begins to form at a z ≈ 20 cm distance. Figure 3a displays a sinusoidal variation of intensity specific to Fresnel zones. The amplitude is modulated with a period that depends more strongly on the distance. A zone period grows proportionally to z^1/2^, while the period of modulations is proportional to *z*. In the first modulation period, the highest contrast is achieved. Theoretical images are complex, and they contain many Fresnel zones. However, insufficient coherence levels might result in a simple pattern having only the first Fresnel zone. We demonstrate this by comparing Figure 3b and Figure 1b.

## 4. Conclusions

Phase-contrast images allow one to estimate the size of micro-pores in crystals. In the near field region of object-to-detector distances *z*, the transverse dimension of a micro-pore can be directly determined as the distance between the deep interference minima positions in the experimental pattern. We have obtained a useful estimate for the distance z, making it possible, and verified it by computer simulations. Namely, *z* should be approximately 10 times less than *z*_c_, which is determined from condition 2(*λ z*_c_)^1/2^ = *D*, where *λ* is the wavelength, and *D* is the size. For a micropipe as an example, *z* = 0.1 cm if the micropipe diameter *D* = 2 μm and *λ* = 0.775 Å. The distance *z* is minimal, and the detector should be very close to the sample.

However, the problem is that micro-pores with a transverse size of less than 2 µm are too small to detect under the above condition. Magnification is needed. It is possible in the far-field region but, under these conditions, simulated patterns are composed of many Fresnel zones absent in experimental results. The CCD camera can detect only the region of the first Fresnel zone, and only this region provides information about the pore size.

## Data Availability

Data is contained within the article.
